# Reproductive Challenges in Ruminants Under Heat Stress: A Review of Follicular, Oocyte, and Embryonic Responses

**DOI:** 10.3390/ani15152296

**Published:** 2025-08-06

**Authors:** Danisvânia Ripardo Nascimento, Venância Antonia Nunes Azevedo, Regislane Pinto Ribeiro, Gabrielle de Oliveira Ximenes, Andreza de Aguiar Silva, Efigênia Cordeiro Barbalho, Laryssa Gondim Barrozo, Sueline Cavalcante Chaves, Maria Samires Martins Castro, Erica Costa Marcelino, Leopoldo Rugieri Carvalho Vaz da Silva, André Mariano Batista, José Roberto Viana Silva

**Affiliations:** 1 Laboratory of Biotechnology and Physiology of Reproduction, Federal University of Ceara, Sobral 62041-040, CE, Brazil; danisvania.dr@gmail.com (D.R.N.); venancianunes@gmail.com (V.A.N.A.); regislaneribeiro02@gmail.com (R.P.R.); gabiximenes14@gmail.com (G.d.O.X.); andrezabiomedicina93@gmail.com (A.d.A.S.); eficordeiro@outlook.com (E.C.B.); laryssa_barroso@hotmail.com (L.G.B.); suelinecavalcante@gmail.com (S.C.C.); samireszootecnia@gmail.com (M.S.M.C.); erikamarcelino302010@gmail.com (E.C.M.); leorugieri@hotmail.com (L.R.C.V.d.S.); 2 Laboratory of Biotechniques Applied to Reproduction, Department of Veterinary Medicine, Federal Rural University of Pernambuco, Recife 52171-900, PE, Brazil; andre.batista@ufrpe.br

**Keywords:** follicular development, heat stress, oocyte maturation, reproductive physiology, ruminants

## Abstract

This review aims to discuss how heat stress affects ovarian follicles and oocytes, steroidogenesis, and embryo development in ruminants. The literature shows that quiescent primordial follicles appear to be less susceptible to heat stress, but from the primary follicle stage onwards, they begin to suffer the consequences of heat stress. These adverse effects are exacerbated when the follicles are cultured in vitro. In antral follicles, heat stress reduces granulosa cell viability and proliferation in both in vivo and in vitro models. Oocyte maturation, both nuclear and cytoplasmic, is also compromised, and embryo quality declines under elevated thermal conditions. These effects are linked to intracellular disturbances, including oxidative imbalance, mitochondrial dysfunction, and altered hormonal signaling. The differences between in vivo and in vitro responses reflect the complexity of the biological impact of heat stress and emphasize the protective role of the physiological microenvironment. A better understanding of how heat stress alters the function of ovarian follicles, oocytes, and embryos is crucial. This knowledge is critical to devise effective strategies that mitigate damage, support fertility, and improve outcomes in assisted reproduction for livestock exposed to high environmental temperatures.

## Simple Summary

Heat stress is the result of the interaction of several climatic variables, including the intensity of solar radiation, air movement, ambient temperature, and humidity. The rise in environmental temperatures has intensified concerns about the negative impact of heat stress on ruminant reproductive performance. This study examines the impacts of thermally unfavorable environments on ovarian follicles, oocytes, hormone production, and embryonic development in ruminants. The data showed that early follicles, from the primary stage onward, suffer the consequences of heat stress, resulting in oocytes with low competence to be fertilized. In antral follicles, the reduction in hormone levels caused by heat negatively affects growth and disrupts the activity of enzymes and proteins responsible for steroid synthesis. With regard to embryonic development, exposure to high temperatures has been associated with a decline in embryo quality. Therefore, developing adaptive mechanisms and thermal management strategies is essential for preserving reproductive efficiency and ensuring the sustainability of animal production under adverse environmental conditions.

## 1. Introduction

Heat stress results from a multifactorial interaction between climatic variables, including the intensity of solar radiation, air movement, ambient temperature, and relative humidity [1,2]. The combination of these environmental factors promotes thermal discomfort in animals, increasing the heat load on their bodies. This compromises their thermoregulatory ability, resulting in inefficient heat distribution and an increase in internal body temperature beyond normal physiological limits [1,2,3,4,5,6,7].

Although different species show varying levels of thermotolerance, heat stress generally manifests itself at environmental temperatures above 30 °C, leading to limited grazing and increased dependence on shade and water sources [6,8,9,10]. In addition, it triggers biochemical and molecular changes in an attempt by the body to compensate for the excessive accumulation of heat [3,7,11]. When exposed to extreme thermal conditions, animals show reduced productivity, which generates economic losses estimated at billions of dollars annually [4,12], associated with significant drops in reproductive performance [13,14].

With the continuous increase in global temperatures, the negative effects of heat stress on ruminant fertility have become a growing cause for concern [9,12,15,16,17]. Structures that are essential for reproductive aspects in ruminants are affected by heat stress. Although in vitro studies have shown that secondary follicles are more sensitive to heat stress than primordial and primary follicles, there is still no clear conclusion about the influence of heat stress on early follicles and its consequences for reproduction in ruminants. This knowledge gap limits our understanding of the quality of oocytes from ovulatory follicles that developed from early follicles exposed to heat stress. In large antral follicles, the reduction in FSH and LH caused by heat negatively affects the growth and survival of these structures in vivo, causing damage to the granulosa cells [18,19]. In vitro studies have also shown that heat stress interferes with enzymes and proteins involved in the synthesis of steroid hormones [20]. In addition, it impairs oocyte quality both in vivo and in vitro [11,21], reduces protein synthesis, and can generate epigenetic modifications [11]. In relation to embryonic development, high temperatures have been associated with a decline in both quality and blastocyst rates in vitro [22,23].

In the context of the consequences of thermal overload on reproduction, this review article aims to discuss the effects of thermal stress during follicular growth from primordial to late antral follicles on steroidogenesis, oocyte maturation, and embryonic development in ruminants.

## 2. Effects of Heat Stress on Early Follicles

**Figure 1:**
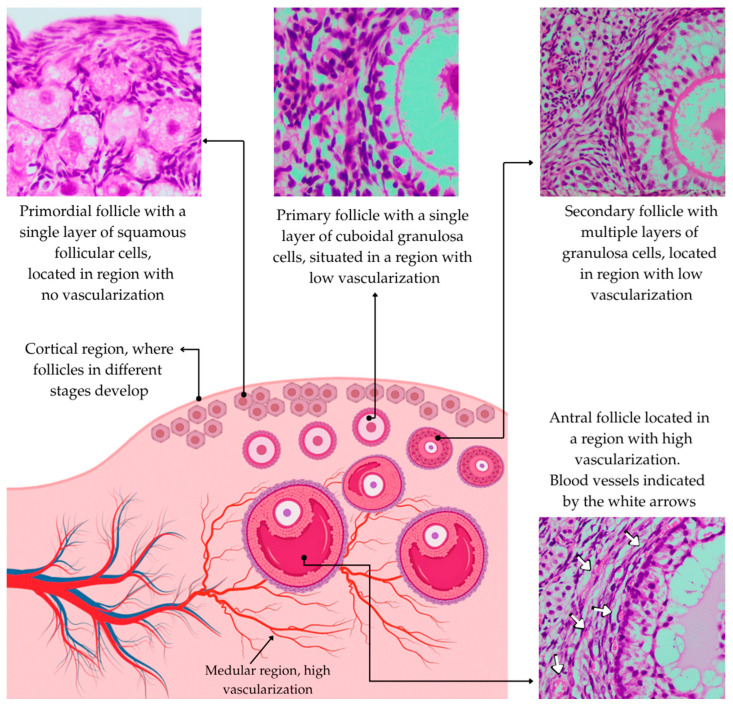
Cortical and medullary regions of the ovary showing that primordial, primary, and secondary follicles are located in regions with low vascularization, whereas antral follicles have well-developed blood vessels in the theca cell layer.

Heat stress is a critical factor affecting ruminant reproductive physiology and can impact endocrine pathways. It leads to increased cortisol and corticotropin-releasing hormone levels [14], resulting in alterations in ovarian hormone production and secretion. This hormonal change inhibits the production of GnRH and deregulates FSH and LH secretion, negatively impacting reproductive efficiency [24,25]. Heat stress can also influence FSH secretion, leading to an increase that is associated with a greater number of developing follicles in the ovary [26]. This increase is likely due to a reduction in inhibin concentration [25]. Stefanska et al. [24] showed a relationship between heat stress and decreased fertility rates, associated with reduced LH and increased FSH. Regarding early follicles, it is well known that quiescent primordial follicles are located in regions of the ovarian cortex with low vascularization (Figure 1) and, consequently, do not directly depend on FSH and LH to grow and survive [27]. This anatomical characteristic may contribute to the resistance of this follicular stage to heat stress, suggesting that, even under adverse conditions, these primordial follicles may not be severely affected. Torres-Júnior et al. [28] demonstrated that the developmental competence of oocytes recovered from cows exposed to heat stress was lower than that of oocytes recovered from control cows for up to 105 days after the end of the stress period. Given that the development of primordial to preovulatory follicles takes around 120 days [29,30] and that of primary follicles to ovulation takes 100 days [31], it can be inferred that oocytes from primary follicles onwards are susceptible to heat stress.

When early follicles are removed from their physiological environment and subjected to in vitro culture, they appear much more sensitive to heat stress. Aguiar et al. [9] reported that all stages of follicular development (primordial, primary, and secondary follicles) are susceptible to heat stress, particularly secondary follicles. The elevated expression of BCL-2 Associated X Protein (BAX) and Superoxide Dismutase 1 (SOD1) genes in these follicles suggests an increase in oxidative stress and apoptosis in response due to heat stress, which could be associated with a reduction in ovarian reserves and reproductive efficiency. Exposure to heat stress for two hours in vitro can negatively impact early follicles [32], causing a significant reduction in primordial follicles. Additionally, morphological assessments revealed a decreased follicle diameter and increased apoptotic granulosa cells, suggesting that heat stress adversely affects both follicular quantity and cellular viability. Despite this sensitivity, early follicles cultured in vitro may demonstrate an adaptive response to heat stress by maintaining the expression of essential genes for cell survival, such as heat shock protein 70 (HSP70), BAX, and myeloid cell leukemia 1 (MCL1) [33]. Granulosa cells play a crucial role in this process, providing additional support by protecting oocytes from heat and oxidative stress. In vitro, granulosa cells of early follicles with additional layers help protect oocytes in these stages by producing antioxidants, such as glutathione and superoxide dismutase, which neutralize free radicals generated by heat stress [34]. The antral follicles cultured under in vivo conditions exhibit sensitivity to heat stress in both in vivo and in vitro environments [35]. Paes et al. [33] highlighted that isolated secondary follicles exhibit greater resistance to heat stress than primordial and primary follicles in vitro.

The difference in sensitivity to heat stress between early follicles in vivo and in vitro may be explained by the fact that early follicles in vivo are protected by the ovarian stroma, the extracellular matrix, stable oxygenation, and paracrine signals, which may reduce oxidative damage [36]. Additionally, factors such as blood flow, nutrient supply, and physiological thermoregulation contribute to modulating the effects of heat stress [37]. In contrast, these protective mechanisms are absent in vitro, resulting in direct and potentially more severe heat exposure [38]. Taken together, these findings suggest that in vitro systems may overestimate the sensitivity of early follicles, as demonstrated by Cardone [32].

## 3. Effects of Heat Stress on Antral Follicles

The formation of the antral cavity in cattle is observed when the follicles reach approximately 300 µm in diameter. Initially located close to the surface of the ovary, a less vascularized region, these follicles migrate towards the medulla as they grow, which coincides with a progressive increase in local vascularization and in the expression of gonadotropin receptors, such as FSHR and LHR, favoring their development [39]. In this context, the differentiation of granulosa cells plays a crucial role in the response to gonadotropins, creating an environment conducive to oocyte development [40]. In addition, cellular communication through transzonal projections, essential for the exchange of nutrients and signals, allows the oocyte to maintain its viability and acquire development capacity [41]. Early antral follicles represent a critical phase of follicular development. At this stage, the oocytes have not yet acquired full competence for maturation and fertilization but are in a process of active growth and intense interaction with cumulus and granulosa cells [42]. This oocyte incompetence in early antral follicles occurs due to the low expression of genes that activate essential signaling pathways to increase their sensitivity to increased gonadotropins [43]. Thus, responsiveness to gonadotropins is essential for follicles to continue their growth until the selection and dominance phase. Considering that these follicles have well-developed vascularization and present increased expression of FSH and LH receptors, they become particularly susceptible to damage caused by heat stress, which compromises ovarian perfusion and can inhibit granulosa cell function, suppressing the progression of follicular development. In this sense, a recent study that combined in vivo and in vitro approaches in goats exposed to 36 °C and 70% humidity for 48 h demonstrated that follicles recruited under heat stress (≥2 mm) showed a significant reduction in the expression of LH receptors, aromatase activity and estradiol production, mainly in medium follicles (3.5–4.9 mm), resulting in follicular regression before ovulation and delayed follicular dynamics, which highlights the negative impact of heat on the steroidogenic and ovulatory competence of follicles [20]. Thus, heat stress directly interferes with the integration between vascularization, hormonal signaling, and oocyte competence, resulting in significant damage to follicular dynamics and fertility. In the context of in vitro culture, the sensitivity of antral follicles to external factors represents a significant challenge. Follicular development between approximately 0.3 µm and 1.0 mm is especially sensitive to adverse handling and environmental conditions, such as heat stress, which can compromise oocyte quality and follicular dynamics [41]. An increase in the depletion of essential amino acids, such as isoleucine, leucine, and valine, was observed in the in vitro follicular environment under heat stress, which may compromise protein synthesis and the energy production required for early antral follicle maintenance. The reduction in alanine consumption also suggests an unfavorable metabolic adaptation, potentially influencing somatic cell functionality and the quality of the follicular microenvironment [41]. Furthermore, the viability of granulosa cells, responsible for providing metabolic support to the oocyte, may be compromised, hindering the progression of follicular development [44]. The combination of these factors may result in a lower ovulation rate and reduced fertility during periods of heat [41].

**Figure 2:**
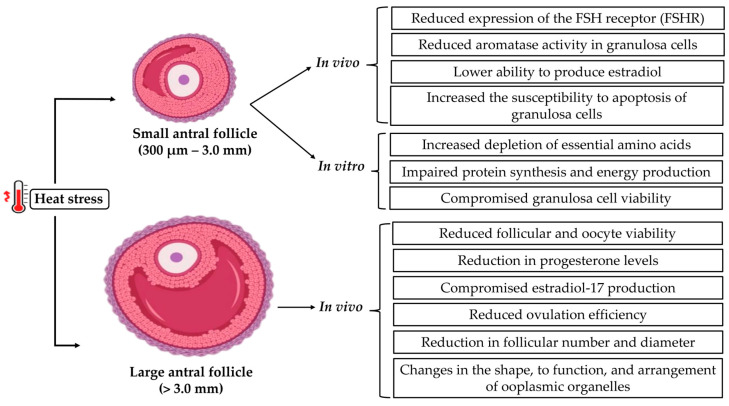
The impact of heat stress on the viability, steroidogenesis, gene expression, and organelles of small and large antral follicles in vivo and in vitro.

Antral follicles above 3 mm have greater vascularization, which makes them more susceptible to stress factors compared to initial antral follicles [45]. However, in vivo studies with antral follicles (3–7 mm in diameter) subjected to heat stress have demonstrated impaired steroidogenesis of theca and granulosa cells, decreased viability of early antral follicles, as well as of the oocytes present in them, reduced progesterone levels, increased risk of early embryonic death, impaired production of estradiol-17, which is essential for ovulation and follicle quality, and decreased secretion of LH, reducing ovulation and the efficiency of artificial insemination, which may result in a lower chance of successful fertilization and development of healthy embryos [18,19]. For in vivo bovine models, it was also observed that with each increase in the temperature–humidity index (THI), this follicular category can decrease in diameter on the day of estrus, in addition to causing damage to the cytoplasm, resulting in changes in the shape, function, and arrangement of ooplasmic organelles, especially mitochondria [46]. Furthermore, studies have reported that exposure to heat can also lead to a reduction in the number of follicles and reduced diameter of surviving follicles, in addition to negatively affecting granulosa cells, essential for follicular development [47]. Heat stress causes changes in early initial and large antral follicles as shown in Figure 2.

## 4. Effects of Heat Stress on Steroidogenic Cells and Pathways

As the primary steroidogenic cells of ovarian follicles, granulosa and theca cells regulate oocyte development and maturation by secreting estrogen and critical growth factors [48]. These cells synthesize progesterone and estradiol [49] through the action of various essential enzymes. The steroidogenic acute regulatory protein (STAR) transports cholesterol into mitochondria, initiating steroid biosynthesis in theca cells [50,51]. CYP11A1 encodes the cholesterol side-chain cleavage enzyme, which converts cholesterol into pregnenolone, the precursor of all steroid hormones, making it an essential enzyme for the biosynthesis of steroid hormones [52,53]. CYP17A1 and CYP19A1 (aromatase) are involved in converting intermediates into androgens and estrogens, respectively, important for estradiol production [54,55]. Theca cells convert cholesterol into androstenedione, whereas granulosa cells aromatize androgens into estradiol [56]. Heat stress disrupts steroidogenesis by impairing the activity and viability of both granulosa and theca cells, leading to reproductive dysfunction across various species, including cattle [57,58]. It also disrupts the cell cycle progression, inhibiting their proliferation [59], consequently disrupting steroid hormone production. Holstein Friesian cows exposed to high temperatures and a THI above 80 during the summer had compromised steroidogenesis caused by reducing LH secretion. This leads to decreased estradiol production, a hormone essential for follicular development and estrus expression [60]. Similarly in Pantanal cows, chronic heat stress with THI > 75 for 60 days also suppressed steroidogenesis in theca and granulosa cells, leading to decreased estradiol production [61].

While in vivo studies provide findings regarding the systemic effects of heat stress, in vitro models allow the investigation of cellular and molecular responses in steroidogenic cells. In vitro culture of buffalo granulosa cells resulted in reduced viability when exposed to 41.5 °C, along with a significant decrease in the concentration of estradiol and progesterone levels [62]. Notably, even when cholesterol metabolism remains unaffected, these cells still exhibited impaired steroidogenic capacity, indicating that heat stress disrupts multiple molecular pathways. Key genes involved in protein protection (HSP), apoptosis (Tumor protein, TP53, and Cyclin-Dependent Kinase Inhibitor, CDKN), and energy metabolism (glycogen phosphorylase, PYGM, alcohol dehydrogenase 6, ADH6, and Solute carrier family 2 member 4, SLC2A4) were dysregulated, affecting glucose metabolism and energy production and ultimately compromising steroidogenesis [63]. Additionally, Li et al. [64] revealed heat-induced alterations in gene expression related to follicular development and cell survival by transcriptome alterations. Specifically, heat stress activated caspase-3, a key executioner of apoptosis, and disrupted the balance between pro-apoptotic BAX and anti-apoptotic BCL-2 proteins. This shift toward cell death pathways not only compromises granulosa cell viability but also contributes to suppressed estrogen biosynthesis, highlighting the close connection between apoptosis and hormone production under thermal stress. Table 1 shows the effect of heat stress on the reduction in progesterone and estradiol levels in several studies.

Regarding the mechanisms involved in the reduction in estradiol levels under heat stress, the sterol regulatory binding proteins (SREBPs)/mevalonate kinase (MVK)-LHR pathway plays a significant role. Ovine granulosa cells cultured in vitro under heat stress showed increased expression of SREBPs and MVK, enzymes that regulate cholesterol biosynthesis. However, this overactivation leads to the formation of the MVK-LHR complex, which promotes the degradation of luteinizing hormone receptor (LHR) mRNA [66]. Since LHR is essential for the LH-mediated stimulation of steroidogenesis in granulosa cells [67], its downregulation results in decreased estradiol synthesis. Other studies have demonstrated the role of the enzyme heme oxygenase (HO-1) and its product carbon monoxide in protecting granulosa cells against heat stress. According to Wang et al. [68], HO-1 expression under heat stress plays a cytoprotective role, reducing apoptosis and helping maintain estradiol levels. When HO-1 is silenced, apoptosis increases and estrogen production declines. On the other hand, Zhu et al. [69] demonstrated that excessive HO-1 expression may have detrimental effects, increasing intracellular iron levels and promoting lipid peroxidation, a hallmark of ferroptosis, which compromise granulosa cell viability and steroid hormone synthesis. Transcriptomic analyses in Turpan Black sheep under heat stress conditions revealed the significant dysregulation of genes involved in ovarian steroidogenesis, including CYP17A1, which encodes the 17α-hydroxylase/17,20-lyase enzyme. This enzyme is responsible for converting progesterone into androgens, which are then aromatized into estrogens. Disruption of CYP17A1 impairs this conversion and directly limits estrogen production. In addition, heat stress altered peroxisomal fatty acid β-oxidation [54]. These alterations suggest that heat stress impacts not only hormone synthesis but also energy metabolism and lipid balance in ovarian cells, reinforcing the role of heat stress in impairing steroid hormone synthesis at the transcriptional level.

Heat stress also disrupts the endoplasmic reticulum function, a cellular organelle responsible for protein folding and processing. Under thermal stress, misfolded proteins accumulate in the endoplasmic reticulum, triggering the unfolded protein response (UPR) in bovine granulosa cells [70]. This response includes the upregulation of Glucose-Regulated Proteins (GRP78 and GRP94), molecular chaperones that serve as markers of endoplasmic reticulum stress [71]. According to Alemu et al. [57], 24 h of heat stress upregulated the expression of mRNA for GRP78 and GRP94, which are endoplasmic reticulum stress markers in bovine granulosa cells. Prolonged endoplasmic reticulum stress disturbs cellular homeostasis and impairs steroidogenic capacity.

In short, the main mechanisms by which heat stress impairs granulosa cells include the following: the decreased expression of essential genes for steroidogenesis (STAR, CYP11A1, CYP17A1, and CYP19A1), LH receptor degradation mediated by the SREBP/MVK pathway, activation of apoptotic pathways (TP53, caspase-3, and BAX/BCL-2), and disruption of energy and lipid metabolism through the downregulation of genes such as PYGM, ADH6, SLCA4, Acyl-CoA oxidase 1- ACOX1, and Fatty acid desaturase 2- FADS2. Additionally, there is the activation of cellular stress responses, such as HSP proteins and endoplasmic reticulum chaperones (GRP78 and GRP94), as well as the involvement of the HO-1/CO pathway, which can be either protective or deleterious. These interconnected mechanisms lead to reduced estradiol and progesterone production and, consequently, decreased fertility in animals exposed to heat stress.

## 5. Heat Stress Affects Oocyte Maturation

Oocyte maturation is fundamental for the success of embryonic development. At this stage, several factors can be crucial for the oocyte to achieve the quality that will support the initial stages of embryonic development. Among the challenges, heat stress directly impacts oocyte quality [72,73]. In vivo studies have demonstrated that heat stress has a detrimental impact on bovine oocyte maturation, with a prolonged effect that often requires several weeks for full recovery due to the lengthy folliculogenesis process [74]. Lee et al. [75] reported that Jersey cows exhibit a higher degree of heat tolerance in relation to their reproductive performance when compared to Holstein cows. Oocytes from heat-stressed Holstein cows produced higher levels of ROS than those from Jersey cows. This excess ROS may cause cytoplasmic damage and abnormal chromosomal segregation during the summer, as the disruption of cellular homeostasis can compromise DNA integrity and induce apoptosis. Additionally, Diaz et al. [21] reported that heat stress (THI above 75) negatively impacts both the quality and quantity of grade 1 bovine oocytes.

In vitro investigations have revealed that heat stress negatively impacts protein production and affects oocyte development and competence by decreasing its maturation rates when it prevents the resumption of meiosis II, consequently directly impacting embryonic development rates [14,72]. Exposure of bovine oocytes to high temperatures (41 °C) in vitro has a negative influence on competence and subsequent embryonic development due to changes in mitochondrial function, affecting adenosine triphosphate (ATP) synthesis and redox potential (ROS levels). This is accompanied by decreased expression of microtubule-associated protein light chain 3 (LC3) and sirtuin 1 (SIRT1), which regulate autophagy and mitochondrial function, respectively [76]. Heat stress also alters amino acid metabolism in the bovine oocyte–cumulus complex, with increased release of phenylalanine, valine, alanine, and glutamic acid and decreases in the levels of leucine, isoleucine, serine, and glutamine, leading to reduced cleavage and blastocyst formation rates [77]. According to Klabnik et al. [78], exposure of bovine oocytes to 41 °C for 2 h has been demonstrated to have direct consequences on the cumulus–oocyte complex and on transcript abundance. Related to cell junctions, plasma membrane rafts, and cell-cycle regulation, potentially influencing meiotic resumption and progression. In prepubescent sheep, Serra et al. [79] reported that extreme environmental temperatures impair oocyte development by compromising cytoplasmic maturation, reducing mitochondrial activity, increasing ROS levels, and causing alterations in the meiotic spindle and chromosomal alignment.

At the molecular level, Diaz et al. [21] identified 211 and 92 differentially expressed genes in germinal vesicle (GV) and metaphase II (MII) oocytes, respectively, as a result of heat exposure. The primary biological processes affected in GV oocytes include steroid biosynthesis, oxidation–reduction reactions, and mitochondrial depolarization. Regulation of the mitogen-activated protein kinase (MAPK) cascade was negatively affected in MII oocytes. MII-stage bovine oocytes collected during summer had a total of 1068 differentially expressed genes, such as Heat Shock Protein (HSPA8), COP9 signalosome subunit 5 (COPS5), RNA Polymerase II, I, and III Subunit L (POLR2L), and Proteasome 26S Subunit, ATPase 6 (PSMC6) being highly ranked [80]. Furthermore, in vitro maturation of ovine oocytes at 42 °C resulted in a reduced proportion of oocytes reaching the MII stage (34.7%) and upregulation of BAX, C-myc, Caspase 3, and P53, as well as decreased expression of BCL-2 [81]. It was also revealed that ovine oocytes collected in the warm season exhibit reduced developmental competence but are more resistant to heat stress in vitro, which may be an adaptation induced by chronic heat stress in vivo [82]. This consequence comes from several oocyte structures and genes affected by heat stress, as seen in Table 2.

## 6. Heat Stress and Embryonic Development

Heat stress is a factor contributing to economic losses, since high ambient temperatures have been associated with decreased in vitro embryo quality or even inhibited embryo development [22,23], resulting from its deleterious effects on embryos across multiple levels, including morphology, biochemistry, transcription, and development [87]. The development of healthy embryos in vitro occurs through the rigid regulation of cellular processes that combine the genetic and cellular components of eggs and sperm during fertilization [88,89]. In bovine species, embryos at the early stages of cleavage (before the 8- to 16-cell stage) present an inactive genome and consequently are more susceptible to various stress factors, including high temperatures. Diaz et al. [87] reported that in vivo or in vitro developing early embryos responded equally to heat stress. A series of cytoplasmic changes occurs during the development of early embryos, including the reorganization of the cytoskeleton, the precipitation of chromatin, the migration of organelles to the center of blastomeres, and the occurrence of mitochondrial edema. Around the third day of development, the embryo reaches the eight to sixteen cell stage, and then activation of the embryonic genome occurs. This is a process in which transcripts derived from oocytes undergo degradation, and in vitro embryo gene transcription initiates to drive further development [88,89].

High temperatures are directly linked to the production of ROS, which mediates oxidative stress and negatively affects various biological functions in early embryos. When an imbalance occurs between the production of ROS and the antioxidant capacity in cells, embryo quality is reduced. Morales-Cruz et al. [90] evaluated the effects of the harvest month and THI on the production of in vitro embryos in Holstein cows and heifers and showed that, in cows, embryo production declined sharply when THI reached 80 units, whereas in heifers, there was a decline in embryo production from THI = 78. Rosales-Martínez et al. [91] observed that the number of degenerated embryos was higher in the hot and dry season (31.6 °C) when compared to other seasons. In sheep, a lower percentage of blastocysts after in vitro fertilization of oocytes was observed in the summer period, and these blastocysts exhibited a significantly higher degree of DNA fragmentation compared to those produced in the winter [92]. In addition, the severity of damage caused by heat stress was related to high temperatures, demonstrating that extreme temperatures significantly impaired oocyte quality and embryo production.

Embryos have protective mechanisms against environmental thermal changes. For instance, heat shock proteins (HSPs) assist cells in adapting to higher temperatures by aiding in the proper folding of misfolded or unfolded proteins caused by cellular stress. The HSP family comprises a variety of types, including HSP110, HSP100, HSP90, HSP70, HSP60, HSP40, HSP27, and HSP10 [93,94,95,96,97,98]. In addition, the expression of HSPs depends on the magnitude of the stress stimulus; for example, HSP70 normally increases its expression in heat exposures greater than 3 °C above normal temperature during short periods of time. In this context, several genes are activated in the face of a variety of stressful stimuli, including cyclooxygenase 1 and 2 (COX1 and COX2), X-linked inhibitor of apoptosis (XIAP), and BCL-2 [99]. In Malabari goats, Amitha et al. [100] evaluated the impact of heat stress on the expression of different genes and reported that heat stress significantly influenced the expression pattern of COX2. Other responses are activated due to cellular stress, such as glycosylation activity, apoptotic, and autophagy mechanisms, which help to remove damaged cells. When none of these mechanisms are controlled, there are severe effects on the reproductive system [95]. Heat shock during in vitro oocyte maturation can disturb the expression of some genes in embryos at the eight-cell stage, and, consequently, alterations in the accumulation of histone H3 lysine 9 trimethylation (H3K9me3) and heterochromatin protein 1 (HP1) can exert a deleterious influence on chromatin remodeling, thereby compromising embryo development and quality [101]. This is evidenced by a diminished blastocyst rate and an augmented apoptotic index. Stamperna et al. [102] demonstrated that elevated temperatures compromise the in vitro developmental potential of early embryos. It was observed that HSP70 can mitigate the deleterious effects of heat stress, thereby enhancing embryonic yield and reducing the adverse effects of heat stress on certain embryo quality characteristics. Faheem et al. [103] evaluated the in vitro embryonic development competence of buffaloes subjected to heat shock. They reported that the initial cellular defense mechanism against heat stress is triggered by the expression of HSPs. These proteins function as the primary line of defense for embryos, helping to maintain cellular redox homeostasis and resist the threats posed by heat stress. Consequently, the enhancement of heat-shock-related genes (HSF1 and HSP90) through transcription can be regarded as a viable approach to augment embryonic thermotolerance.

**Figure 3:**
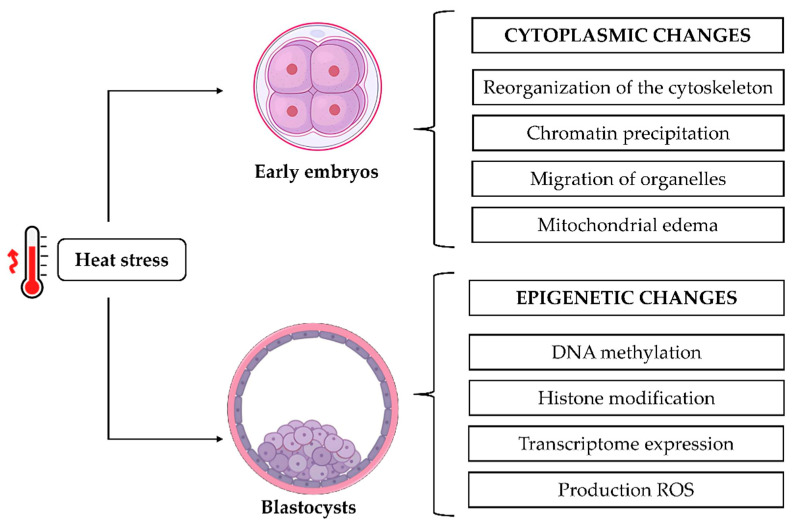
The effects of heat stress on the cytoplasm, chromatin, transcription, and ROS levels during embryonic development.

Exposure to elevated temperatures can also trigger epigenetic alterations in cells, including DNA methylation and histone modifications, which can subsequently impact the expression of genes within the genome [97]. Furthermore, these epigenetic changes can influence the expression of HSP70, a protein that functions as a heat stress biomarker [98]. When exposed to thermal stress, the expression of this protein increases thermotolerance at the molecular level. Heat stress has been demonstrated to reduce the expression of epigenetically related genes, including DNA methyltransferase 1 (DNMT1), DNMT3A, DNMT3B, and histone H2A, and to decrease the developmental capacity of blastocysts [11]. It has been posited that heat stress in pregnant cows may induce epigenetic modifications in the developing embryo germ cells. These modifications, in turn, have the potential to induce phenotypic effects in offspring. The study demonstrated a significant association between the month of birth, season of pregnancy, and heat stress index of F0 females with the performance of their F2 and F3 progenies. This finding suggests a true transgenerational effect [104]. Heat stress causes cytoplasmic changes and epigenetic modifications during embryonic development, as shown in Figure 3.

## 7. Final Considerations

From the primary stage onwards, follicles suffer the consequences of heat stress, and the effects on oocyte quality can be observed up to 100 days after thermal stress. The consequences of heat-induced oxidative damage are numerous and include alterations in gene expression, reduced oocyte competence, disturbances in granulosa cell function, diminished steroid hormone production, and poor embryo quality. The progeny of the exposed generation can experience adverse effects due to defects in chromatin remodeling and epigenetic changes caused by heat stress. The resultant alterations are responsible for reductions in meat and milk yields, which in turn engender substantial economic losses for the livestock industry. The comprehension of the molecular and biochemical pathways influenced by heat stress in ovarian follicles, steroidogenesis, oocytes, and embryos is imperative to engineer interventions that safeguard animal reproductive performance.

Considering the current climate changes, the development and implementation of integrated strategies for the management of livestock along with the genetic enhancement of thermotolerance are of paramount importance. Additionally, the refinement of in vitro embryo production technology with respect to thermal physiology can facilitate the production of high-quality embryos. It is imperative to acknowledge the significance of these actions in preserving the reproductive capacity of ruminants.

## 8. Conclusions

Early ovarian follicles, from the primary stage onward, experience the effects of heat stress, resulting in oocytes with low competence to be fertilized and to assure embryo development. Heat stress reduces the production of gonadotropins, which negatively affects oocyte growth and disrupts the activity of enzymes responsible for steroidogenesis in granulosa and theca cells. Furthermore, exposure to high temperatures has been linked to a decline in embryo quality in ruminants.

## Figures and Tables

**Table 1:** The effect of heat stress on ovarian steroidogenesis.

Experimental Condition	Effects on Steroidogenesis
Bovine granulosa cells cultured at 40 °C and 41 °C	Heat stress caused a 45% reduction in progesterone production at both temperatures, and estradiol levels decreased by 60%, with a more pronounced reduction at 40–41 °C. This was associated with the downregulation of CYP11A1 and STAR, increased reactive oxygen species (ROS), and mitochondrial dysfunction [57].
Cows exposed to heat for 30 and 60 days before ovum pick-up (OPU)	Progesterone production decreased due to LH alteration. Estradiol production and follicular development declined, with altered gonadotropins (increased FSH, decreased LH), reduced oocyte viability, and decreased Bone Morphogenetic Protein (BMP15) and Growth Differentiation Factor 9 (GDF9) [60].
Pantaneira and Girolando cows exposed to heat stress for 60 days	Chronic heat stress reduced oocyte viability in Pantaneira cows due to decreased estradiol production. Girolando cows maintained oocyte competence through efficient thermoregulation under tropical conditions [61].
Bovine granulosa cells cultured in vitro at 43 °C	Progesterone and estradiol production had significant reductions. There were 256 differentially expressed genes (DEGs) and 51 altered metabolites, impacting Transforming Growth Factor Beta (TGF-β) and Vascular Endothelial Growth Factor (VEGF) pathways and modifying amino acid metabolism [65].
Bovine granulosa cells cultured in vitro at 43 °C	Progesterone and estradiol concentrations decreased. There were 330 DEGs (75 upregulated, 255 downregulated), increased ROS and apoptosis, and alterations in the TP53 and Regulatory Associated Protein of mTOR Complex 1 (RPTOR) pathways, indicating metabolic reprogramming [63]

**Table 2:** Adverse effects of heat stress on oocytes of ruminants.

Experimental Condition	Damage Caused by Heat Stress
Oocytes cultured in vitro at 41 °C for 12 h and then at 38.5 °C in bovine species	DNA methylation and DNA hydroxymethylation Reduced ATP levels Reduced mitochondrial distribution and mitochondrial DNA copies Reduced oocyte transzonal projections Reduced in mRNA expression of GDF9, BMP15, and Mitogen-activated protein kinase 1- MAPK1 [11]
Oocytes cultured in vitro under moderate high (40 °C) and low (37 °C) stress in bovine species	Impaired embryonic development rates [83]
Bovine oocytes cultured in vitro at 41 °C	Reduced transzonal projections [78]
Oocytes cultured in vitro from 38.5 °C to 40.5 °C (for 6 h) and reduced again to 38.5 °C in bovine species	Decreased expression of Interferon Tau and increased reactive oxygen species [23]
Bovine oocytes cultured in vitro (41 °C for 12 h)	Disrupted the abundance of transcripts from bta-miR-19b and DROSHA and impaired embryonic development rates [72]
Bovine oocytes cultured in vivo (THI over 75)	Negative effects on the quality and number of health oocytes (Grade I); differentially expressed genes in oocyte at GV and MII stages [75]
Bovine oocytes cultured in vitro at 40.5 °C	Depletion/appearance of amino acids [77]
Ovine oocytes cultured in vitro at 41 °C	Abnormal chromatin configurations and reduced cleavage rates [84]
Ovine oocytes cultured in vitro at 42 °C	Increased expression of BAX, C-myc, Caspase 3, and P53 and decreased expression of BCL-2 [81]
Ovine oocytes cultured in vitro at 41 °C for 12 h and then at 39 °C	Reduced cleavage and blastocyst formation rates [82]
Ovine oocytes cultured in vitro at 41 °C	Altered the expression patterns of interleukin (IL-6) and its receptor [85]
Caprine oocytes cultured in vitro at 41 °C	Reduced maturation rates and blastocyst rates [86]

## Data Availability

Further information on the data included in this study is available from the corresponding author.
